# Supplementary material for: End Sequence Analysis Toolkit (ESAT) expands the extractable information from single-cell RNA-seq data
Source: Genome Res. 2016 Oct;26(10):1397–410. doi: 10.1101/gr.207902.116 (PMC5052061; doi:10.1101/gr.207902.116)
Supplement: Supplemental Material [file supp_gr.207902.116_Supp_Materials.tar.gz › ESAT-esat_v0.1/doc/html/classbroad_1_1core_1_1sequence_1_1_transcribed_regions.html]

ESAT: broad.core.sequence.TranscribedRegions Class Reference


ESAT


- Main Page
- Related Pages
- Packages
- Classes
- Files

- Class List
- Class Index
- Class Hierarchy
- Class Members

All Classes Namespaces Files Functions Variables Enumerator Pages

- broad
- core
- sequence
- TranscribedRegions

Classes |
Public Member Functions |
Static Public Member Functions |
List of all members

broad.core.sequence.TranscribedRegions Class Reference

|  |  |
| --- | --- |
| Public Member Functions | |
|  | TranscribedRegions (String genomeFasta, String bedFile) throws IOException |
|  | |
| Map< String, Collection< Gene > > | getGenes () |
|  | |
| int | shiftPosition (String chr, int pos, int offset) |
|  | |
| Strand | getOrientation (String chr, int pos) |
|  | |
| char | getTranscribedBase (String chr, int pos, Strand strand) throws IOException |
|  | |
| char | getTranscribedBase (String chr, int pos) throws IOException |
|  | |

|  |  |
| --- | --- |
| Static Public Member Functions | |
| static void | main (String[] args) throws IOException |
|  | |

## Detailed Description

Author
:   prussell

## Constructor & Destructor Documentation

|  |  |  |  |
| --- | --- | --- | --- |
| broad.core.sequence.TranscribedRegions.TranscribedRegions | ( | String | *genomeFasta*, |
|  |  | String | *bedFile* |
|  | ) |  | throws IOException |

Parameters
:   |  |  |
    | --- | --- |
    | genomeFasta | Genome fasta file |
    | bedFile | Bed gene annotation |

Exceptions
:   |  |  |
    | --- | --- |
    | IOException |  |

Here is the caller graph for this function:

## Member Function Documentation

|  |  |  |  |  |
| --- | --- | --- | --- | --- |
| Map<String, Collection<Gene> > broad.core.sequence.TranscribedRegions.getGenes | ( |  | ) |  |

Get the genes

Returns
:   Genes by chromosome

|  |  |  |  |
| --- | --- | --- | --- |
| Strand broad.core.sequence.TranscribedRegions.getOrientation | ( | String | *chr*, |
|  |  | int | *pos* |
|  | ) |  |  |

Get the transcribed orientation of the position based on the gene set Returns unknown if the position does not overlap a gene or strand is ambiguous

Parameters
:   |  |  |
    | --- | --- |
    | chr | Chromosome |
    | pos | Position |

Returns
:   The orientation

Here is the caller graph for this function:

|  |  |  |  |
| --- | --- | --- | --- |
| char broad.core.sequence.TranscribedRegions.getTranscribedBase | ( | String | *chr*, |
|  |  | int | *pos*, |
|  |  | Strand | *strand* |
|  | ) |  | throws IOException |

Get the oriented transcribed base at the position based on orientation provided or based on orientation of overlapping exon if orientation provided is null

Parameters
:   |  |  |
    | --- | --- |
    | chr | Chromosome |
    | pos | Position |
    | strand | Strand or null if get from annotation |

Returns
:   The transcribed base

Exceptions
:   |  |  |
    | --- | --- |
    | IOException |  |

Here is the call graph for this function:

Here is the caller graph for this function:

|  |  |  |  |
| --- | --- | --- | --- |
| char broad.core.sequence.TranscribedRegions.getTranscribedBase | ( | String | *chr*, |
|  |  | int | *pos* |
|  | ) |  | throws IOException |

Get the oriented transcribed base at the position based on orientation of overlapping exon

Parameters
:   |  |  |
    | --- | --- |
    | chr | Chromosome |
    | pos | Position |

Returns
:   The transcribed base

Exceptions
:   |  |  |
    | --- | --- |
    | IOException |  |

Here is the call graph for this function:

|  |  |  |  |  |  |
| --- | --- | --- | --- | --- | --- |
| static void broad.core.sequence.TranscribedRegions.main | ( | String[] | *args* | ) | throws IOException |

static

Parameters
:   |  |  |
    | --- | --- |
    | args |  |

Exceptions
:   |  |  |
    | --- | --- |
    | IOException |  |

Here is the call graph for this function:

|  |  |  |  |
| --- | --- | --- | --- |
| int broad.core.sequence.TranscribedRegions.shiftPosition | ( | String | *chr*, |
|  |  | int | *pos*, |
|  |  | int | *offset* |
|  | ) |  |  |

Shift a position along the direction of transcription If the shifted position is not in the same block as the original position, this method currently throws an exception Throws an exception if the position is not in any transcribed interval

Parameters
:   |  |  |
    | --- | --- |
    | chr | Chromosome |
    | pos | Original position |
    | offset | Offset along direction of transcription (can be negative) |

Returns
:   Shifted position within the transcript

---

The documentation for this class was generated from the following file:

- java/broad/core/sequence/TranscribedRegions.java


---

Generated on Fri Aug 15 2014 15:10:56 for ESAT by  

 1.8.7
